# Female genital mutilation and skilled birth attendance among women in sub-Saharan Africa

**DOI:** 10.1186/s12905-021-01578-w

**Published:** 2022-01-30

**Authors:** Abdul-Aziz Seidu, Richard Gyan Aboagye, Barbara Sakyi, Collins Adu, Edward Kwabena Ameyaw, Joycelyn Boatemaa Affum, Bright Opoku Ahinkorah

**Affiliations:** 1 Centre for Gender and Advocacy, Takoradi Technical University, P.O. Box 256, Takoradi, Ghana; 2 Department of Estate Management, Takoradi Technical University, P.O. Box 256, Takoradi, Ghana; 3 College of Public Health, Medical and Veterinary Sciences, James Cook University, Townsville, QLD 4811 Australia; 4 Department of Family and Community Health, School of Public Health, University of Health and Allied Sciences, Ho, Ghana; 5 Department of Population and Health, University of Cape Coast, Cape Coast, Ghana; 6 Department of Health Promotion, Education and Disability Studies, Kwame Nkrumah University of Science and Technology, Kumasi, Ghana; 7 School of Public Health, Faculty of Health, University of Technology Sydney, Sydney, Australia

**Keywords:** Female genital mutilation, Skilled birth attendance, Sub-Saharan Africa, Women’s health

## Abstract

**Background:**

There is evidence that women who have had their genitals cut suffer substantial difficulties during and/or after childbirth, including the need for a caesarean section, an episiotomy, an extended hospital stay, post-partum bleeding, and maternal fatalities. Whether or not women in sub-Saharan Africa who have undergone female genital mutilation utilize the services of skilled birth attendants during childbirth is unknown. Hence, we examined the association between female genital mutilation and skilled birth attendance in sub-Saharan Africa.

**Methods:**

The data for this study were compiled from 10 sub-Saharan African countries’ most recent Demographic and Health Surveys. In the end, we looked at 57,994 women between the ages of 15 and 49. The association between female genital mutilation and skilled birth attendance was investigated using both fixed and random effects models.

**Results:**

Female genital mutilation and skilled birth attendance were found to be prevalent in 68.8% and 58.5% of women in sub-Saharan Africa, respectively. Women with a history of female genital mutilation had reduced odds of using skilled birth attendance (aOR = 0.91, 95% CI = 0.86–0.96) than those who had not been circumcised. In Ethiopia, Guinea, Liberia, Kenya, Nigeria, Senegal, and Togo, women with female genital mutilation had reduced odds of having a trained delivery attendant compared to women in Burkina Faso.

**Conclusion:**

This study shed light on the link between female genital mutilation and skilled birth attendance among sub-Saharan African women. The study's findings provide relevant information to government agencies dealing with gender, children, and social protection, allowing them to design specific interventions to prevent female genital mutilation, which is linked to non-use of skilled birth attendance. Also, health education which focuses on childbearing women and their partners are necessary in enhancing awareness about the significance of skilled birth attendance and the health consequences of female genital mutilation.

## Background

Approximately 63 million girls are likely to be circumcised by 2050 [1], making it a global concern. All surgeries that entail partial or total removal of the external female genitalia for cultural reasons are included in this practise [2]. Female genital mutilation (FGM) is common in 30 African countries, with an estimated three million girls at risk of FGM each year [1, 3]. FGM is prevalent not just in Africa, but also in Asia and other European nations where migrants from FGM-practicing communities reside [2]. FGM is a societal phenomenon in some regions of Africa, linked to social mores and religion, and justified by the preservation of virginity, which is a requirement for marriage, initiation ceremonies, identity, conjugal fidelity, honour, purity, and increased fertility [2, 4, 5]. Many contextual factors, such as highly unequal cultures in which gender prescriptions demand girls' virginity before marriage, have been proven to reinforce FGM. The gender perspective of FGM is also rooted in the socio-cultural norm that emphasises the need for men to control women’s sexuality, prevent promiscuity, ensure premarital virginity, marital fidelity and male sexual satisfaction. This is often being widely considered as a result of patriarchal oppression and the subjugation of women [6].

The World Health Organization (WHO) classifies FGM into four categories: Types 1 and 2, also known as clitoridectomy and excision, respectively, involve the partial or complete removal of the clitoris and labia, while Type 3 involves cutting and repositioning of the labia to create a partial cover and may involve stitching the tissues together (this is the most radical of all the procedures), and finally, Type 4 involves piercing or scraping of the genitalia [3]. According to the 2016 United Nations Children's Fund (UNICEF) report, around 90% of FGM cases include either Types 1 (mainly clitoridectomy), 2 (excision), or 4 (“nicking” without flesh removed), and about 10% (over 8 million women) have gone through Type 3 (infibulation) [1].

According to a 2016 UNICEF report, Types 1 (primarily clitoridectomy), 2 (excision), and 4 (“nicking” without flesh removed) account for around 90% of FGM incidences, while Type 3 (infibulation) accounts for about 10% (about 8 million women) [1]. Infibulation, the most severe form of FGM, is predominantly practised in Djibouti, Eritrea, Ethiopia, Somalia, and Sudan in the North-Eastern area of Africa. The tendency in West Africa (Guinea, Mali, Burkina Faso, and so on) is to remove flesh (clitoridectomy and/or excision) without sewing the labia minora and/or majora together. The high prevalence of infibulation in Africa's North-East area has a negative impact on birthing [2]. High maternal mortality and FGM prevalence have long been critical concerns of public health interest in many countries in sub-Saharan Africa (SSA) [7].

The majority of high-FGM-prevalent nations also have high maternal mortality ratios and high numbers of maternal fatalities, with SSA accounting for approximately 66% of maternal deaths in 2017 [7]. The increased prevalence of maternal mortality and FGM has the opportunity to decelerate the achievement of Sustainable Development Goals 3 and 5 by 2030.

FGM has been shown in numerous studies to be more of a disadvantage than a benefit to mutilated women. It has been linked to a variety of consequences, including extreme pain, haemorrhage, infection, cyst formation, keloids, sexual dysfunction, chronic pelvic infection, obstetric issues, and death [1–3, 8, 9]. FGM causes major difficulties during labour, including the need for a caesarean section, an episiotomy, and a protracted hospital stay, as well as post-partum bleeding and maternal fatalities [8–11]. Scar formation as a result of FGM is one of the ways in which the procedure exposes women to complications after childbirth [8]. These scars cause vaginal stenosis, in which the vaginal walls fail to gradually dilate, putting the baby's and mother's lives at danger of morbidity and mortality [8, 9].

Women who have experienced FGM are much more likely to encounter difficulties during childbirth and their newborns are more likely to die as a result of the procedure, according to studies on FGM and obstetric outcome in SSA [2, 10]. This is especially true for women who have had infibulation since they are more likely to experience protracted and obstructed labour, which can lead to foetal mortality and obstetric fistula [10].

Previous research on FGM and maternal healthcare utilization (antenatal care services) found that in a non-normative community, women who have experienced FGM may find it difficult to socialize with others [12]. It’s possible that this is linked to stigma and discrimination. According to Goffman [13], stigma is a social feature that transforms a person “from a whole and ordinary person to a tainted, devalued one.” As a result, physical differences caused by FGM may expose women who have already undergone it to stigma, relegating them to the status of ‘other’ in the greater social context, potentially preventing them from accessing public spaces. Women who have been subjected to FGM are also more likely to reject negative socio-cultural norms and practices that serve as a barrier to the utilization of maternal healthcare services, such as skilled birth attendance (SBA).

Given the difficulties associated with FGM and childbirth, circumcised women are undeniably at risk of morbidity and mortality during delivery, and this risk is exacerbated for the majority of circumcised women who deliver outside of a hospital or obstetric setting and are supervised by unskilled birth attendants [10]. Regardless, no research has been conducted to determine if women who have experienced FGM are more or less likely to use skilled birth attendants during delivery. This is significant because skilful delivery may safeguard women who have been subjected to FGM during childbirth and lower their odds of dying during the delivery process. In this regard, we examined the association between FGM and SBA in 10 countries in SSA, where data on FGM and other important variables considered in this study are available. This study is based on the hypothesis that women who have undergone FGM will be less likely to utilise the services of skilled birth attendants compared to those who have not undergone FGM.

## Methods

### Study design

The data for this study were compiled from the most recent Demographic and Health Surveys (DHS) conducted in 10 sub-Saharan African countries between 2010 and 2020 (Table 1). DHS is a globally representative and comparative survey done in over 85 low- and middle-income countries [14]. The survey is conducted by the MEASURE DHS with support from organisations within the various countries such as the statistical service and the Ministry of Health. DHS employed a cross-sectional design which was carried out descriptively to collect data from the respondents. The survey used a two-stage cluster sampling technique to recruit respondents. The first stage of sampling consisted of compiling a list of primary sampling units (PSUs) or enumeration areas (EAs) that covered the entire country which was obtained from the most recent national census. The EAs were further subdivided into standardized segments of between 100–500 households each. A random sample of a predetermined segment was later chosen with a probability proportional to the size of the EA. In the second stage, households were systematically selected from a list of previously enumerated households in each selected EA segment and those who were usual residents of selected households or visitors who slept in the households on the night before the survey are interviewed. The study collected data from respondents on health indicators such as mother and child health, sexual and reproductive health, domestic violence, and men's health using a cross-sectional approach [14]. The data was collected from the participants using a standardised questionnaire. A recent work [15] has provided elaborate information on the DHS’ sampling and data collection procedure. A total of 175,870 women aged 15–49 years from the 10 countries were extracted for the study. Out of this, 57,994 of the women who had a childbirth history within five years prior to the survey and also had complete observations on all their corresponding variables were included in the final analysis. Thus, all the women with missing observations were dropped. In writing this paper, we followed the STROBE (Strengthening Reporting of Observational Studies in Epidemiology) reporting criteria [16]. The dataset is freely and publicly available for download at https://dhsprogram.com/data/available-datasets.cfm. All methods were performed in accordance with the Helsinki Declarations and guidelines.

**Table 1:** Description of the study sample

SN, country	Year of survey	Weighted N	Weighted %
1. Burkina Faso	2010	10,438	18.0
2. Ethiopia	2016	3482	6.0
3. Guinea	2018	5459	9.4
4. Kenya	2014	6674	11.5
5. Liberia	2019–2020	3374	5.8
6. Mali	2018	3225	5.6
7. Nigeria	2018	7715	13.3
8. Sierra Leone	2019	7056	12.2
9. Senegal	2010–2011	7105	12.2
10. Togo	2013–2014	3466	6.0
All countries	–	57,994	100.0

### Variables studied

#### Outcome variable

SBA was considered as the outcome variable. The question "Who assisted [NAME] during delivery?" was used to analyse this variable. This was the most recent birth of the mother within the five year period prior to the survey. “Traditional Birth Attendant/Others” and “Skilled birth attendants/Health professionals” were the two responding categories. This classification and categorisation has been used in previous DHS research [17–20].

#### Key explanatory variable

FGM was the key explanatory variable. To obtain this variable, the respondents were asked if they had undergone FGM. “Have not undergone FGM” and “Have undergone FGM” were the response possibilities. The existing coding of the replies in the DHS datasets was retained and used in the final study and was in line with previous literature [21].

### Covariates

The study included a total of 15 variables as covariates. These variables were divided into two categories: individual and contextual factors. The factors were chosen based on their availability in the DHS dataset as well as prior studies' substantial associations with SBA [17–20].

#### Individual factors

The individual factors included maternal age, educational level, marital status, working status, religion, antenatal care (ANC) attendance, media exposure (television, radio, and newspaper), difficulty getting permission to go to the health facility, difficulty getting money needed for treatment, and difficulty with distance to the health facility. We kept the existing coding for all the variables except ANC attendance, religion, and exposure to media. ANC attendance was recoded as no visit, one to three visits, and four or more visits. Christianity, Islam, African Traditional religion, No religion, and others were used to recode religion. Those who said “not at all” to radio, newspaper, or television were categorised as “not exposed [No],” whereas those who said “yes” were categorised as “exposed [Yes].” In each of the three variables, this categorisation was applied (radio, newspaper, and television).

#### Contextual factors

Contextual factors were wealth index, place of residence, and countries studied. We used the DHS dataset's current coding for wealth index and location of residence. The wealth index was divided into five categories: poorest, poorer, middle, richer, and richest. Residence was categorised as either “urban” or “rural.” As part of the contextual factors, all of the countries investigated were taken into account.

### Statistical analyses

**Fig. 1:**
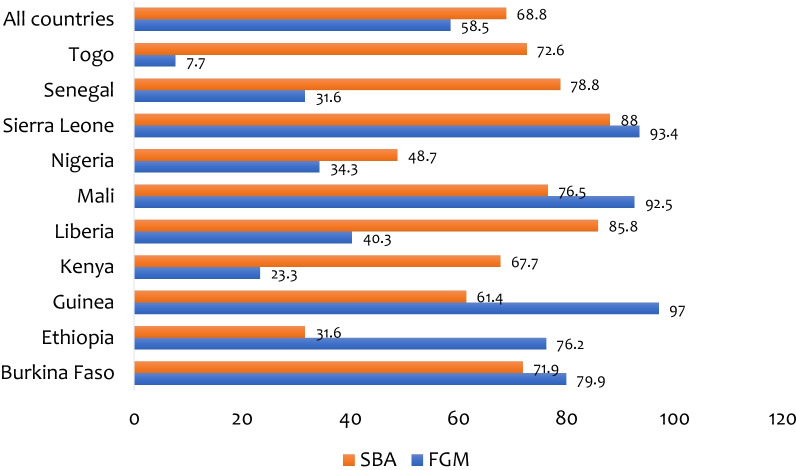
Prevalence of FGM and SBA among women in sub-Saharan Africa

Stata software version 16.0 was used to analyse the data (Stata Corporation, College Station, TX, USA). The analysis was carried out in three steps. To present the results of the prevalence of FGM and SBA in the first stage, percentages were employed (Fig. 1). Second, Pearson's chi-square test was used to evaluate the distribution of SBA across the explanatory variables with a p-value less than 0.05 showing statistical significance. Later, using four models, a multilevel binary logistic regression analysis was done to assess the relationship between FGM and SBA while controlling for individual and contextual characteristics. Without the explanatory variables, Model 0 indicated the variance in SBA ascribed to the clustering of the PSUs. The key explanatory variable and the individual characteristics were included in Model I. The contextual factors are included in Model II. The key explanatory variable, as well as individual and contextual factors, were included in the final model (Model III). These models were fitted using the Stata command “melogit.” For model comparison, we employed Akaike's Information Criterion (AIC) tests. The fixed effect model was adopted for this study due to the complex data structure of the DHS dataset. Also, the fixed effect model took care of the variations between higher level units and lower level units within the data structure [22]. The results were presented as adjusted odds ratios (aOR) with a 95% confidence interval (CI). To improve the generalizability of our findings, we employed sample weight (v005/1,000,000) and the ‘svy' command to cater for over and under-sampling, including the complicated survey design.

### Ethical considerations

Since our research was based on publicly available data, we did not seek ethical approval for this work. Ethical approval was obtained prior to the start of the survey, and all ethical criteria governing the use of human participants were properly followed. Details about data and ethical standards are available at: http://goo.gl/ny8T6X.

## Results

### Prevalence of FGM and SBA among women in sub-Saharan Africa

The overall prevalence of FGM and SBA among women in SSA were 68.8% and 58.5% respectively. At the country level, the highest prevalence of FGM was 97% in Guinea, with the lowest prevalence in Togo (32.5%). On the other hand, the highest prevalence of SBA was 88% in Sierra Leone, with the lowest prevalence in Ethiopia (31.6%) (see Fig. 1).

### Distribution of SBA across FGM and other characteristics of women in sub-Saharan Africa

Table 2 presents the results on the bivariate analysis of FGM and SBA among women in SSA. The results showed a significant disparity in FGM and SBA*.* Specifically, SBA was less prevalent among women who had undergone FGM (67.8%) compared to those who had never undergone FGM (70.3%). All the covariates showed significant associations with SBA.

**Table 2:** Distribution of SBA across FGM and other characteristics of women in sub-Saharan Africa

Variables	Weighted N	Weighted %	SBA Yes (%)	*p* value
*FGM status*				0.005
Not undergone FGM	24,056	41.5	70.3	
Undergone FGM	33,938	58.5	67.8	
*Maternal age*				< 0.001
15–19	3941	6.8	71.1	
20–24	12,294	21.2	71.0	
25–29	15,208	26.2	70.4	
30–34	11,805	20.4	67.6	
35–39	8887	15.3	67.7	
40–44	4300	7.4	64.0	
45–49	1558	2.7	59.9	
*Educational level*				< 0.001
No education	32,210	55.6	59.8	
Primary	12,579	21.7	71.2	
Secondary	10,985	18.9	87.2	
Higher	2220	3.8	95.6	
*Marital status*				< 0.001
Never married	3482	6.0	86.6	
Married	48,405	83.5	66.6	
Cohabiting	3581	6.2	81.1	
Widowed	702	1.2	61.6	
Divorced	650	1.1	67.3	
Separated	1174	2.0	77.0	
*Religion*				< 0.001
Christianity	20,888	36.0	74.1	
Islam	34,817	60.0	66.7	
African Traditional	1481	2.6	48.0	
No religion	748	1.3	62.2	
Others	59	0.1	53.4	
*Current working status*				< 0.001
No	20,106	34.7	64.0	
Yes	37,888	65.3	71.4	
*Antenatal care attendance*				< 0.001
None	5664	9.8	16.5	
1–3 times	20,240	34.9	62.9	
4 or more times	32,090	55.3	81.8	
*Difficulty getting permission to go to the health facility *				< 0.001
Big problem	10,990	19.0	63.8	
Not a big problem	47,004	81.0	70.0	
*Difficulty getting money needed for treatment*				< 0.001
Big problem	33,278	57.4	64.3	
Not a big problem	24,716	42.6	75.0	
*Difficulty with distance to the health facility*				< 0.001
Big problem	21,968	37.9	57.3	
Not a big problem	36,026	62.1	75.9	
*Exposure to television*				< 0.001
No	34,468	59.4	58.6	
Yes	23,526	40.6	83.8	
*Exposure to radio*				< 0.001
No	21,525	37.1	59.7	
Yes	36,469	62.9	74.3	
*Exposure to newspaper/magazine*				< 0.001
No	51,564	88.9	66.4	
Yes	6430	11.1	88.8	
*Wealth index*				< 0.001
Poorest	12,075	20.8	45.2	
Poorer	11,799	20.4	57.8	
Middle	11,638	20.1	68.3	
Richer	11,737	20.2	82.2	
Richest	10,744	18.5	93.5	
*Place of residence*				< 0.001
Urban	19,817	34.2	88.7	
Rural	38,177	65.8	58.5	

### Multilevel regression analysis of the association between FGM and SBA among women in sub-Saharan Africa

Table 3 shows the multilevel regression analysis of the association between FGM and SBA among women in SSA. After adjusting for all covariates, we found that women who had undergone FGM (aOR = 0.91, 95% CI = 0.86–0.96) were less likely to utilize SBA compared to those who had not undergone FGM. At the country level, in Ethiopia, Guinea, Liberia, Kenya, and Nigeria, Senegal, and Togo, women who have had FGM had reduced odds of SBA compared to women in Burkina Faso. With the covariates, maternal age, educational level, marital status, working status, religion, ANC attendance, difficulty with permission to visit the health facility, difficulty with distance to the health facility, exposure to television and newspaper/magazine, wealth index and place of residence had significant associations with SBA.

**Table 3:** Mixed-effects analysis of the association between FGM and SBA among women in sub-Saharan Africa

Variable	Model o	Model I Adjusted odds ratios [95% CI]	Model II Adjusted odds ratios [95% CI]	Model III Adjusted odds ratios [95% CI]
*Fixed effect results*				
*FGM*				
Not undergone FGM		Reference category		Reference category
Undergone FGM		1.43*** [1.36, 1.49]		0.91** [0.86, 0.96]
*Maternal age*				
15–19		Reference category		Reference category
20–24		0.86, 1.04]		50. [0.81, 0.99]
25–29		0.88** [0.80, 0.97]		0.84*** [0.76, 0.92]
30–34		0.86** [0.78, 0.95]		0.81*** [0.73, 0.90]
35–39		0.90* [0.81, 0.99]		0.86** [0.78, 0.96]
40–44		0.85** [0.76, 0.95]		0.85** [0.76, 0.96]
45–49		0.81** [0.70, 0.94]		0.82* [0.70, 0.95]
*Maternal educational level*				
No education		Reference category		Reference category
Primary		1.20*** [1.13, 1.27]		1.41*** [1.32, 1.50]
Secondary		2.28*** [2.11, 2.47]		2.08*** [1.91, 2.27]
Higher		5.75*** [4.54, 7.30]		4.68*** [3.67, 5.97]
*Marital status*				
Never married		Reference category		Reference category
Married		0.57*** [0.51, 0.64]		0.84** [0.74, 0.95]
Cohabiting		0.79** [0.68, 0.91]		0.87 [0.74, 1.01]
Widowed		0.49*** [0.40, 0.60]		0.72** [0.58, 0.90]
Divorced		0.49*** [0.39, 0.61]		0.86 [0.68, 1.09]
Separated		0.66*** [0.55, 0.80]		0.84 [0.69, 1.03]
*Current working status*				
No		Reference category		Reference category
Yes		1.30*** [1.25, 1.37]		1.12*** [1.06, 1.17]
*Religion*				
Others		Reference category		Reference category
Christianity		3.37*** [1.68, 6.77]		2.57* [1.24, 5.31]
Islamic		2.96** [1.47, 5.94]		1.69 [0.82, 3.49]
African Traditional		2.13* [1.05, 4.33]		1.40 [0.67, 2.92]
No religion		2.62** [1.28, 5.38]		2.28* [1.08, 4.80]
*Antenatal care attendance*				
0		Reference category		Reference category
1–3		7.72*** [7.09, 8.41]		5.52*** [5.03, 6.06]
4 or more		16.3*** [14.98, 17.77]		10.67*** [9.72, 11.72]
*Difficulty with permission to go the health facility*				
Big problem		Reference category		Reference category
Not a big problem		0.73*** [0.69, 0.77]		0.81*** [0.76, 0.86]
*Difficulty getting money needed for treatment*				
Big problem		Reference category		Reference category
Not a big problem		0.96 [0.92, 1.01]		1.00 [0.95, 1.06]
*Difficulty with distance to health facility*				
Big problem		Reference category		Reference category
Not a big problem		1.88*** [1.79, 1.97]		1.63*** [1.55, 1.72]
*Exposure to television*				
No		Reference category		Reference category
Yes		2.15*** [2.04, 2.26]		1.35*** [1.27, 1.43]
*Exposure to radio*				
No		Reference category		Reference category
Yes		1.17*** [1.12, 1.23]		0.99 [0.94, 1.04]
*Exposure to newspaper/magazine*				
No		Reference category		Reference category
Yes		1.32*** [1.20, 1.46]		1.32*** [1.19, 1.46]
*Wealth index*				
Poorest			Reference category	Reference category
Poorer			1.80*** [1.71, 1.91]	1.41*** [1.33, 1.50]
Middle			2.85*** [2.68, 3.02]	1.90*** [1.78, 2.02]
Richer			5.56*** [5.17, 5.99]	2.89*** [2.67, 3.14]
Richest			15.15*** [13.59, 16.90]	5.06*** [4.48, 5.71]
*Place of residence*				
Urban			Reference category	Reference category
Rural			0.42*** [0.39, 0.45]	0.51*** [0.47, 0.54]
*Countries*				
Burkina Faso			Reference category	Reference category
Ethiopia			0.15*** [0.14, 0.17]	0.17*** [0.15, 0.19]
Guinea			0.47*** [0.43, 0.51]	0.52*** [0.47, 0.57]
Kenya			0.51*** [0.46, 0.55]	0.20*** [0.18, 0.22]
Liberia			2.22*** [1.98, 2.47]	0.76*** [0.66, 0.87]
Mali			0.99 [0.88, 1.10]	1.23** [1.08, 1.39]
Nigeria			0.25*** [0.23, 0.27]	0.14*** [0.13, 0.16]
Sierra Leone			3.08*** [2.81, 3.38]	1.63*** [1.47, 1.81]
Senegal			1.05 [0.97, 1.14]	0.85** [0.78, 0.94]
Togo			0.69*** [0.62, 0.76]	0.39*** [0.35, 0.44]
*Random effect results*				
Primary Sampling unit variance (95% CI)	0.739 [0.655, 0.834]	0.559 [0.488, 0.640]	0.368 [0.319, 0.425]	0.252 [0.214, 0.296]
Intra-class correlation coefficient	0.183	0.145	0.101	0.071
Likelihood ratio test	3067.99 (< 0.001)	1644.02 (< 0.001)	1350.75 (< 0.001)	810.92 (< 0.001)
Wald chi-square	Reference	8768.67***	9256.01***	11,069.75***
*Model fitness*				
Log-likelihood	− 35,171.908	− 28,535.782	− 28,289.52	− 25,531.563
Akaike’s Information Criterion	70,347.82	57,131.56	56,611.04	51,151.13
Sample size	57,994	57,994	57,994	57,994
Number of clusters	1609	1609	1609	1609

Exponentiated coefficients; 95% confidence intervals in brackets; CI Confidence Interval

*p < 0.05, **p < 0.01, ***p < 0.001

## Discussion

FGM is a social phenomenon in SSA that is linked to social mores and religion, and is justified by the preservation of virginity, which is a requirement for marriage, initiation ceremonies, identity, marital fidelity, honour, purity, increased fertility, and hygiene [2, 4, 5]. Using the most recent DHS dataset, we looked at the association between FGM and SBA among women in SSA from 2010 to 2020. The prevalence of SBA was 58.5%, with Sierra Leone (88%) having the highest prevalence and Ethiopia having the lowest (31.6%). We discovered a 68.8% prevalence of FGM among women in SSA, with Guinea (97%) having the greatest and Togo having the lowest (7.7%). The prevalence of SBA in this study was substantially higher than in earlier studies conducted in Bangladesh (35.9%) [23] and Nepal (48%) [24]. Furthermore, the prevalence of FGM was higher than the 61% reported in a prior Ghanaian study [25].

Women who had undergone FGM were less likely than those who had not undergone FGM to use SBA services in this study. The stigma associated with FGM may explain this outcome, as it makes women unwilling to seek SBA services. We believe that the decreased prevalence of SBA services among women who have had FGM may be explained by perceived stigmatization in formal medical settings where there are no regulations addressing their unique SBA services, as reported by Goffman [13]. FGM typically occurs in traditional settings where the practice is widespread, but women who have had FGM may become aware of the 'otherness' of their bodies outside of these settings, which can cause discomfort and exclusion when seeking SBA services in healthcare facilities. In a previous study, women who have undergone FGM are faced with lack of specialized understanding of their health needs and also being treated by male healthcare providers may cause discomfort [26]. Other scholars have indicated that breach of privacy and confidentiality within the healthcare setting hinders FGM victims' utilisation of maternal health services of which SBA is a major component [27, 28]. Due to the prohibition of FGM in countries in SSA such as Kenya, Nigeria, Guinea, Liberia, and Burkina Faso among others, FGM victims fear their status can cause arrest and legal actions against their relatives, hence, their preference not to seek health services including SBA [27, 28].

It was found that women who had 4 or more antenatal care visits had higher likelihood of obtaining SBA at birth as compared to women who had no antenatal care visits. Previous research has found similar results in Ethiopia [29], Sierra Leone, Niger, and Mali [30], Egypt [31], and Zambia [32]. It is likely because women who attend ANC are informed about the necessity of expert birth attendance. As a result, women may develop a behavioural change towards expert delivery support during their ANC follow-up.

This study also discovered residential diversity in the use of SBA. Quality services, lack of qualified personnel, poor socio-economic position, and a variety of socio-cultural norms of women may limit SBA use in rural areas compared to their urban counterparts. Longer distances to reach healthcare facilities, the expenses of maternal treatments, and restricted transportation options may all be challenges for rural women [17]. Women's health-promoting behaviour and access to SBA should be boosted by comprehensive community-level interventions that include residential homogeneity in relation to socioeconomic empowerment and infrastructures.

Women with a higher level of education had a higher likelihood of using SBA services than women without any formal education. Previous research in Ethiopia [33, 34], Ghana [35], Guinea-Bissau [36], and Nigeria [37] found that educational attainment was a predictor of SBA service consumption. Higher education has generally been found to be a robust predictor of maternal healthcare consumption [17, 38]. Higher education, according to Dapaah and Nachinaab [39], exposes women to health-related knowledge, which may impact their use of maternity healthcare facilities. According to research, women with some form of formal education are more receptive to innovative health-promoting ideas that support the demand side of health [39]. Several research (e.g. [40–45]) have found that well-educated women have more autonomy in making health-related decisions for themselves and their children.

Wealth index was also found to be a correlate of use of SBA in the study. When compared to their counterparts in the poorest income quintile, women in the highest wealth quintile had a higher likelihood of using maternal healthcare services such as SBA, a finding that is consistent with earlier researches conducted in diverse settings [46, 47]. Due to a lack of financial resources, women from low-income households may prioritise basic daily necessities above healthcare services [48, 49]. Women from wealthy families, on the other hand, may not encounter such obstacles because they have the financial means to pay for professional childbirth. Appropriate service delivery approaches are required in this context to address vulnerable impoverished women groups in society [49]. Despite the association found in all the countries, the differences in maternal and child health policy, education, cultural differences, and existing interventions may affect the results.

### Strengths and limitations

The study's key strength is that it employed nationally representative datasets from the 10 sub-Saharan African countries studied. This is significant because it permits the findings to be applied to all women in the selected countries. The study also made use of sophisticated data analysis methods to ensure that the data was thoroughly examined. Apart from that, the data collection included well-trained field assistants and well-designed questionnaires, resulting in a higher response rate. This ensures that the findings are accurate. Despite these strenghts, the study has several limitations that must be acknowledged. The first drawback is related to the research methodology used. It is worth noting that because this was a cross-sectional study, it could only reveal parameters linked to FGM and SBA among women in SSA, not established causative links. This study could have been hampered by recollection biases, which are common in DHS data. Another limitation is that since the data were collected at different periods, there is, therefore, the possibility of policy changes within and between these countries that could have confounded our results.

## Conclusion

This study sheds light on FGM and SBA in SSA. FGM and SBA were found to be prevalent in SSA. Women with a history of FGM had reduced odds of using SBA than those who had not been circumcised. The parameters linked with FGM and skilful birth delivery in SSA were determined to be maternal age, marital status, exposure to television, exposure to newspapers, wealth index, place of residence, and employment status. These factors provide relevant information to government agencies working on gender, children, and social protection to help them design specific interventions to prevent FGM, which has been linked to poor reproductive health outcomes, and to increase the use of SBA. Also, health education which focuses on childbearing women and their partners are necessary in enhancing awareness about the significance of skilled birth attendance and the health consequences of FGM.

## Data Availability

Data for this study is available at: http://dhsprogram.com/data/available-datasets.cfm.

## Abbreviations

AIC: Akaike’s information criterion
ANC: Antenatal care
aOR: Adjusted odds ratio
CI: Confidence interval
cOR: Crude odds ratio
DHS: Demographic and Health Survey
FGM: Female genital mutilation
PSU: Primary sampling units
SBA: Skilled birth attendance
SSA: Sub-Saharan Africa
WHO: World Health Organisation
